# Curcumin-loaded nanoliposomes linked to homing peptides for integrin targeting and neuropilin-1-mediated internalization

**DOI:** 10.1080/13880209.2016.1261301

**Published:** 2016-12-10

**Authors:** Sogol Kangarlou, Sorour Ramezanpour, Saeed Balalaie, Shahla Roudbar Mohammadi, Ismaeil Haririan

**Affiliations:** a Department of Pharmaceutical Biomaterials School of Pharmacy, Tehran University of Medical Sciences, Tehran, Iran;; b Peptide Chemistry Research Center, K.N. Toosi University of Technology, Tehran, Iran;; c Department of Medical Mycology School of Medical Sciences, Tarbiat Modares University, Tehran, Iran;; d Medical Biomaterials Research Center, Tehran University of Medical Sciences, Tehran, Iran

**Keywords:** Encapsulation, stability, endosome escape, nanotechnology

## Abstract

**Context:** Curcumin, a naturally occurring polyphenol, has been extensively studied for its broad-spectrum anticancer effects. The potential benefits are, however, limited due to its poor water solubility and rapid degradation which result in low bioavailability on administration.

**Objectives:** This study encapsulates curcumin in nanoliposomes including an integrin-homing peptide combined with a C end R neuropilin-1 targeting motif for targeted delivery and receptor-mediated internalization, respectively.

**Materials and methods:** The linear GHHNGR (Glycine–Histidine–Histidine–Asparagine–Glycine–Arginine) was synthesized through F-moc chemistry on 2-chlorotrityl chloride resin and conjugated to oleic acid. The lipoyl-peptide units were then co-assembled with lecithin and 0–75 mole % Tween-80 into liposomes. Curcumin was passively entrapped using a film hydration technique and its degradation profile was examined within seven consecutive days. The cytotoxic effects of the curcumin-loaded liposomes were studied on MCF-7 and MDA-MB-468, during 24 h exposure in MTT assay.

**Results:** The maximum curcumin entrapment (15.5% W/W) and minimum degradation (< 23%) were obtained in a pH switch loading method from 5.7 to 8, in nanoliposomes (< 50 nm) containing oleyl-peptide, lecithin and Tween-80 (1:1:0.75 mole ratio). The oleyl-peptide did not prove any haemolytic activity (< 1.5%) up to 10-fold of its experimental concentration. The curcumin-loaded liposomes displayed significant reduction in the viabilities of MCF-7 (IC_50_ 3.8 μM) and MDA-MB-468 (IC_50_ 5.4 μM).

**Discussion and conclusion:** This study indicated potential advantages of the peptide-conjugated liposomes in drug transport to the cancer cells. This feature might be an outcome of probable interactions between the targeted nanoliposomes with the integrin and neuropilin-1 receptors.

## Introduction

**Figure 1.**
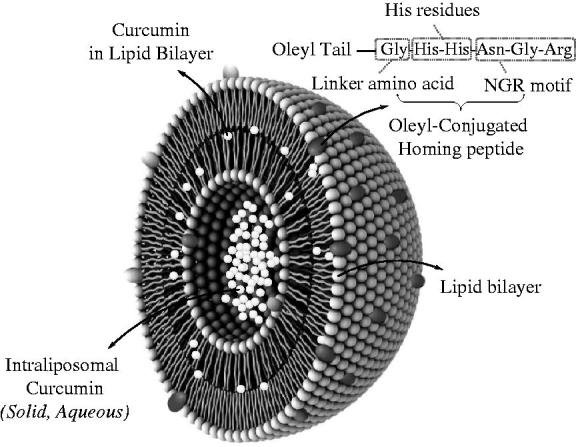
A schematic illustration of the Oleyl-GHHNGR.

Curcumin, a component of turmeric, is one of the naturally bioactive compounds that modulates various biochemical cascades by intervening a diverse range of molecular targets including transcriptional and growth factors, inflammatory cytokines, kinases, adhesion molecules, anti-apoptotic proteins and related receptors (Anand et al. 2008). Accumulating evidence suggests that the curcumin-induced cell death is mediated by the activation of both intrinsic (Chen et al. 2010) and extrinsic (Ak & Gülçin 2008) apoptotic and growth-inhibitory signaling pathways (Bush et al. 2001). In addition to the radical and hydrogen peroxide scavenging, metal chelating and superb antioxidant properties (Baum & Ng 2004); some antitumour effects of curcumin have been attributed to the production of reactive oxygen species (ROS) in transformed cells (McNally et al. 2007; Gandhy et al., 2012). The beneficiary effect has also been reported in numerous diseases and disorders (Aggarwal & Harikumar 2009) as major depressive (Sanmukhani et al. 2014) and neurodegenerative (Baum & Ng 2004), cardiovascular (Li et al. 2008), pulmonary, metabolic (Zhang et al. 2013), hypertriglyceridaemia (Sahebkar et al. 2014), autoimmune (Egan et al. 2004), inflammatory (Khanna et al. 2007; Taylor & Leonard 2011), musculoskeletal (Henrotin et al. 2014), viral (Santo et al. 2003) and neoplastic (Ravindran et al. 2009) diseases. Despite the numerous biological activities, curcumin has not yet been widely introduced in clinical use due to its low water solubility, limited bioavailability (Anand et al. 2007), short half-life and rapid hydrolysis and degradation (Tønnesen 2006). To circumvent these obstacles, various delivery systems were studied among which the liposomes and lipid-like structures conjugated with targeting ligands or antibodies displayed significant improvement in kinetic and dynamic profiles of curcumin (Ranjan et al. 2013). Tumour-homing peptides containing RGD (Marchand-Brynaert et al. 1999) or NGR motifs (Curnis et al. 2006), have been exploited for targeting of therapeutics or diagnostics to cells with an overexpression of α_υ_β integrin family (Avraamides et al. 2008). A class of peptides containing H/K/R/XX/H/K/R (H, K, R indicate histidine, lysine and arginine residues, respectively) at their C-terminus, the so-called C end R, are homologous with the C-terminal domains of VEGF-A165 (Vander Kooi et al. 2007; Ruoslahti et al. 2009). These peptides are reported to induce extravasation via their interaction with neuropilin-1 (Nrp-1) receptors (Teesalu et al. 2009; Haspel et al. 2011) that are overexpressed on several tumour cells such as those derived from breast cancer (Stephenson et al. 2002). Modified peptides and peptidomimetics with C end R domains are potent Nrp-1 antagonists that block the VEGF-Nrp-1 interaction (von Wronski et al. 2006) and improves the internalization of the co-drugs into the tumour cells (Ruoslahti et al. 2010). Recently, a proton sponge effect has been detected for histidine/imidazole-rich peptides, polymers and lipids (Midoux et al. 2009) that is related to the protonation of the secondary and tertiary amines at acidic pH of endosomes (El-Sayed et al. 2009), the fusion of positively charged element to negative inner surfaces, induction of high osmotic pressure and destabilization of the endosomes (Varkouhi et al. 2010). Histidylated carriers have occasionally been used to increase the endosomal escape and intracellular trafficking of different therapeutics (Kichler et al. 2003). In the current assay, a six-residue linear peptide was designed and conjugated to the oleyl tail which was co-formulated into a liposome for encapsulation of curcumin (Figure 1). The cytotoxic properties were further investigated in breast epithelial cancer cells, MCF-7 and MDA-MB-468.

## Materials and methods

### Materials

2-Chlorotrityl chloride (2-CTC) resin and 2-(1H-benzotriazol-1-yl)-1,1,3,3-tetramethyluronium tetrafluoroborate (TBTU) were obtained from GL Biochem. (Shanghai, China). The side-chain protected Fmoc-amino acids were purchased from Zhejiang Materials Industry Co. (Zhejiang, China). *N*-Ethyl diisopropylamine (DIPEA), curcumin, oleic acid, (soy) lecithin, 3-(4,5-dimethylthiazol-2-yl)-2,5-diphenyl tetrazolium bromide (MTT reagent A), polysorbate (Tween) 80 and Triton X-100 were obtained from Merck (Darmstadt, Germany). 2-Chloranil was purchased from Sigma-Aldrich Co (Hamburg, Germany). Dulbecco’s modified eagle medium (DMEM/F-12, GlutaMAX^TM^) was obtained from Gibco^®^, Invitrogen (Waltham, MA).

MCF-7 and MDA-MB-468, poorly invasive/non-metastatic (Thompson et al. 1992; Tong et al. 1999) luminal A (ER^+^, PR±, HER2^−^) (Holliday & Speirs 2011; Voss et al. 2011) and metastatic (Tu et al. 2011) basal (triple-negative) phenotypic human breast adenocarcinoma, respectively, were obtained from Iranian Biological Resource Center (IBRC, Tehran). Heparinized human red blood cell was obtained from Iranian Blood Transfusion Organization (IBTO, Tehran).

### Peptide synthesis, purification and detection

The peptide was synthesized based on the valid protocols of Fmoc chemistry in solid-phase peptide synthesis. Oleic acid was conjugated to the *N*-terminus *N*-deprotected glycine with TBTU and DIPEA. The oleyl-peptide was cleaved from the resin and deprotected with 1% and 95% TFA solutions, respectively. The crude oleyl-peptide was precipitated in diethyl ether and purified in Platinblue preparative HPLC (Knauer, Germany) equipped with a Waters 10 μm, 120 × 20 mm C_18_ preparative column and ChromGate/EZChrom Elite^TM^ (V 3.1.6) acquisition software. The purification was performed using an acetonitrile gradient from 10 to 80% during 30 min with a flow rate of 15 mL min^−1^, at 30 °C and with UV-DAD detection at 220 nm. The mobile phases included acetonitrile and Milli-Q water plus 0.1% TFA. The purity of the fraction(s) was further analyzed with 20 μL injections into a Waters Nova-Pak C_18_, 4 μm, 150 × 3.9 mm (Ireland) analytical column, using similar conditions and with a flow rate of 1 mL min^−1^. The oleyl-peptide fraction was acidified with hydrochloric acid (1 N) to substitute the trifluoroacetate anions in conjunction with arginine residues (Andrushchenko et al. 2007), before lyophilization. The oleyl-peptide mass was confirmed with Agilent 6410 Triple Quadrupole LC/MS (Germany) at 1000 ppm in acetonitrile and Milli-Q water (1:1) with 0.1% TFA with an electrospray ionization mass detector, operated in positive mode and with a fragmentation voltage of 60 V.

### Curcumin-loaded liposome preparation

Dried thin films of curcumin, oleyl-peptide and lecithin were prepared at different mole ratios from their mixtures in chloroform (Table 1). The solvent was evaporated in rotary evaporator followed by storage in vacuum oven at 25 °C overnight. The thin films were then hydrated in two steps by adding Tween-80 followed by sodium phosphate buffer (50 mM, pH 5.7) and 30 s sonication (at 42 kHz) after each step. The mixtures were filtered through 0.45 μm mixed cellulose ester (MCE) membrane to separate the non-entrapped colloidal and precipitated curcumin. In T_5_*_m_* liposomes, NaOH was added to raise the pH to 8 followed by 30 s sonication. The pH returned to 5.7 by drop wise addition of HCl and the suspension was filtered as stated before. The mass and the mole % of the entrapped curcumin were measured with Jasco V-530 UV-Vis spectrophotometer (Tokyo, Japan) at 467 nm (Leung et al. 2008). Immediately before the analysis, the lipid membrane was dispersed with triton X-100 (1% V/V) and the sample was alkalinized to pH 13.

**Table 1.** Liposome composition and % of entrapped curcumin in curcumin-loaded liposomes.

						CM_entrapped_ %^a^	
Sample	[OP] (μg mL^−1^)	[CM] (μg mL^−1^)	[L] (μg mL^−1^)	[T] (μg mL^−1^)	OP:L:T mole ratio	mass (SEM)	mole (SEM)
T_1_	200	100	0	0	1:0:0	0.6 (0.02)	1.5 (0.06)
T_2_	200	100	0	69.6	1:0:0.25	0.8 (0.01)	2.1 (0.04)
T_3_	200	100	0	139.2	1:0:0.5	0.9 (0.01)	2.6 (0.04)
T_4_	200	100	0	208.8	1:0:0.75	1.3 (0.03)	3.8 (0.10)
T_5_	200	100	164.9	208.8	1:1:0.75	2.1 (0.05)	5.5 (0.14)
T_5_*_*m*_*	200	100	164.9	208.8	1:1:0.75	6.4 (0.15)	15.5 (0.32)

OP: oleyl-GHHNGR, MWt. 941.13 (g mole^−1^); CM: curcumin; L: lecithin; T: tween-80.

a SEM = SD/*n*^½^ with *n* = 3 (independent experiments).

### Particle size, zeta charge and morphological study

A methanolic solution of the lyophilized peptide was diluted with sodium phosphate (10 mM, pH 5.5 and 7) or glutamate (10 mM, pH 3) buffers to 40 μg mL^−1^. The size and the charge of the oleyl-peptide particles and the curcumin-loaded liposomes were tested with Brookhaven zetasizer (Holtsville, NY). The particle sizes were corresponded to scanning electron micrographs using Hitachi S-4160 field emission (FE-SEM, Tokyo, Japan). The presence of the bilayer in liposomal curcumin was confirmed after the solvent evaporation in freshly prepared T_5_*_m_* and using Philips CM-30 transmission electron microscope.

### Curcumin degradation kinetics

The curcumin absorbance was measured in T_3-5_, and T_5_*_m_* at pH 7.4 during seven consecutive days, at 24 h intervals and at 25 ± 0.5 °C in triplicate. The samples were stored at −20 °C before analysis. Triton X-100 and NaOH were immediately added before the measurement. The degradation of free curcumin in the aqueous suspension was measured at pH 6.5 during 48 h at 25 ± 0.5 °C. To avoid the loss of curcumin due to gradual precipitation/adsorption in aqueous suspensions, aliquots of stock suspension were taken in separate vials at time zero and each successive time points and stored at −20 °C.

### Haemolysis assay

The plasma fraction was separated from heparinized human whole blood by centrifugation cycles at 4000 *g* followed by washing steps in NaCl (0.9%). The erythrocytes were suspended and diluted in sterile phosphate buffer saline (PBS, pH 7.4, 285 mOsmol kg^−1^) yielding a suspension of 5 × 10^8^ red blood cells (RBC) mL^−1^ (El-Sayed et al. 2005). A stock solution of the oleyl-peptide in PBS (pH 7.4) was diluted within a concentration frame of 0.05–2 mM and mixed with RBCs (10^8^ cells mL^−1^). After 1 h incubation at 37 ± 0.5 °C, the samples were centrifuged at 4000 *g* for 10 min. The absorbance of the released haemoglobin in the supernatant was measured at 410 and 541 nm and compared with the RBCs treated with either PBS or SDS solution (10 mg mL^−1^) as negative or positive controls for 0 or 100% haemolysis, respectively. The haemolytic activity of the oleyl-peptide was calculated by Equation (1):

(1) $$\mathrm{Haemolysis}\;\left(\%\right)=\frac{A_{S}-A_{\mathrm{PBS}}}{A_{\mathrm{SDS}}-A_{\mathrm{PBS}}}\times 100$$

where *A*_S_, *A*_PBS_ and *A*_SDS_ are the absorbance at 410 or 541 nm for the sample, SDS and PBS, respectively.

### In vitro cytotoxicity assay

MCF-7 and MDA-MB-468 were grown in DMEM/F-12, GlutaMax^TM^ at their third passage for 48 h. After trypsination, 1 × 10^4^ cells were seeded in 96-well microplates and incubated at 37 °C, 5% CO_2_ for 24 h. Ethanolic curcumin was diluted in 2.2% glycine, pH 7.4 (Tønnesen 2006) and the pH of the blank and T_5_*_m_* liposomes was adjusted to 7.4 before filtering through 0.22 μm MCE membrane. The concentration of curcumin in glycine and T_5_*_m_* filtrate was measured spectrophotometrically at 467 nm. The samples were serially diluted and different concentrations were added to four wells in triplicate with PBS as the negative control. After 24 h of incubation, MTT solution (5 mg mL^−1^) was added and incubated for 4 h before the medium was replaced with DMSO and sterile Sorenson buffer (5:1). The plate was incubated for 10 min and the absorbance of the formazan salt was measured at 595 nm (545 and 630 nm filters) by Elisa plate reader (Adolf Fenz, Germany). The % of viable cells was calculated by Equation (2):

(2) $$\mathrm{Cell}\;\mathrm{viability}\;\left(\%\right)=\frac{A_{s}-A_{b}}{A_{c}-A_{b}}\times 100\;$$

in which *A*_s_, *A*_b_ and *A*_c_ indicate the absorbance in the sample, blank and the negative control, respectively. The IC_50_ values were determined using the viability % and the median-effect equation/plot in CalcuSyn V2.11, Equation (3):

(3) $$\mathrm{Log}\;(f_{a}/f_{u})=m\;\mathrm{Log}D-m\;\mathrm{Log}D_{m}$$

where *f*_a_, *f*_u_ and *D_m_* indicate the fraction of affected, unaffected and half-affected (potency) cells, respectively. *D* and *m* represent the variable drug dose and the slope (sigmoidity), respectively.

## Results

### Curcumin loading % determination

The % of entrapped curcumin in T_1-5_ and T_5_*_m_* was averaged from three different experiments (Table 1). A reciprocal correlation was observed between the loading % and the mole ratios of Tween-80 in T_1-4_ or the incorporation of lecithin in T_5_. The loading % in T_5_*_m_* displayed a considerable increase comparing to T_5_.

### Particle size, zeta potential and morphology

**Figure 2.**
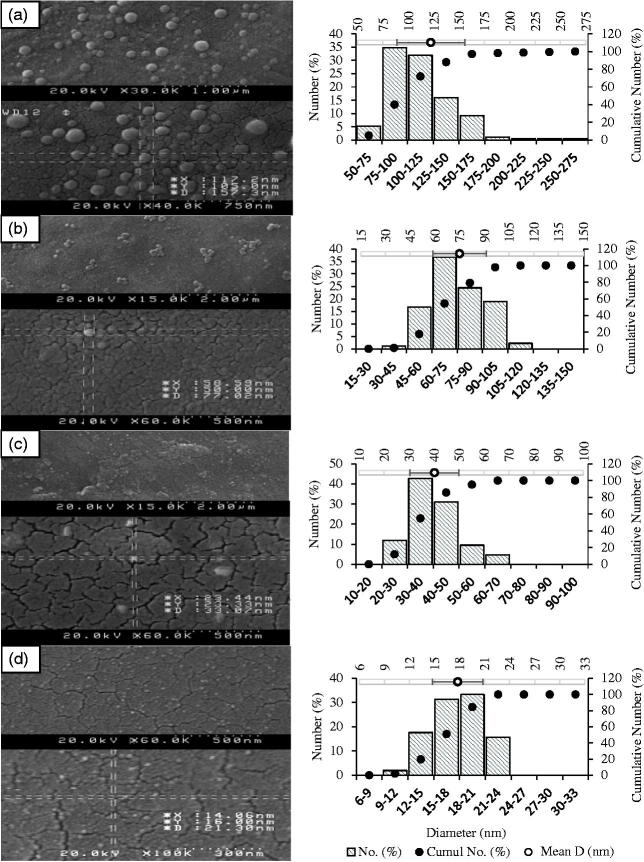
(Left) Scanning electron micrographs for (a) oleyl-peptide in PBS, pH 5.5; (b) T_2_, (c) T_3_ and (d) T_4_ in PBS, pH 5.7; (Right) average diameter and size distribution (ImageJ, 1.49 v, Bethesda, MD).

**Figure 3.**
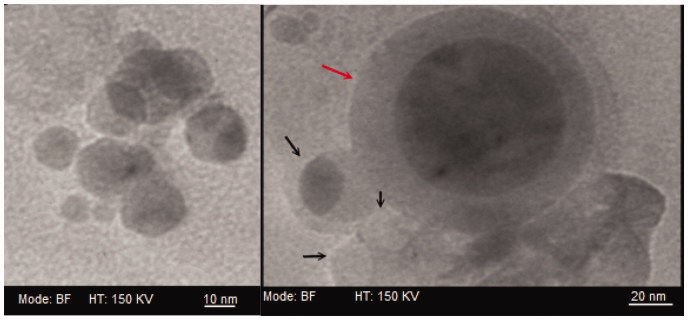
Transmission electron micrographs of a giant/bilamellar (gray arrow) and unilamellar (black arrow) curcumin-loaded T_5_*_m_*-liposomes in PBS, pH 5.7.

According to Table 2, the mean diameter was inversely correlated to the concentration of Tween-80. The zeta charge (*ζ*) in oleyl-peptide suspension displayed a considerable shift (an absolute value of 46 mV) within a pH frame of 3 to 7. The surface charge of the liposomes resembled mainly to those of the peptide particles at neutral pH. Figure 2(a–d) displays the surface micrographs of oleyl-peptide (pH 5.5) and T_2-4_ (pH 5.7). The mean diameter in electron micrographs (ImageJ, 1.49 v, Bethesda, MD) corresponded to the light scattering experiment (122.2 ± 33.5, 75.0 ± 16.2, 40.2 ± 9.8 and 17.8 ± 3.0 nm for oleyl-peptide, T_2_, T_3_ and T_4_, respectively). The formation of the bilayer in T_5_*_m_* (pH 5.7) is shown in Figure 3 with the gray and the black arrows designating a giant or bilamellar and a unilamellar liposome, respectively.

**Table 2.** Particle size and charge measurement.

Sample	pH	Mean diameter (nm)	PDI	*ζ* (mV)
OP	3	106.7	0.293	+20.30
OP	5.5	144.1	0.208	−6.23
OP	7	147.1	0.153	−25.92
T_1_	5.7	ND	ND	ND
T_2_	5.7	75.7	0.160	−4.13
T_3_	5.7	36.2	0.200	−13.53
T_4_	5.7	20.3	0.265	−13.82
T_5_	5.7	16.6	0.253	−12.83
T_5_*_*m*_*	5.7	ND	ND	−13.28
T_5_*_*m*_*	7.4	13.1	0.303	−21.14

PDI: polydispersity index; OP: oleyl-GHHNGR; ND: not determined.

### Curcumin degradation

**Figure 4.**
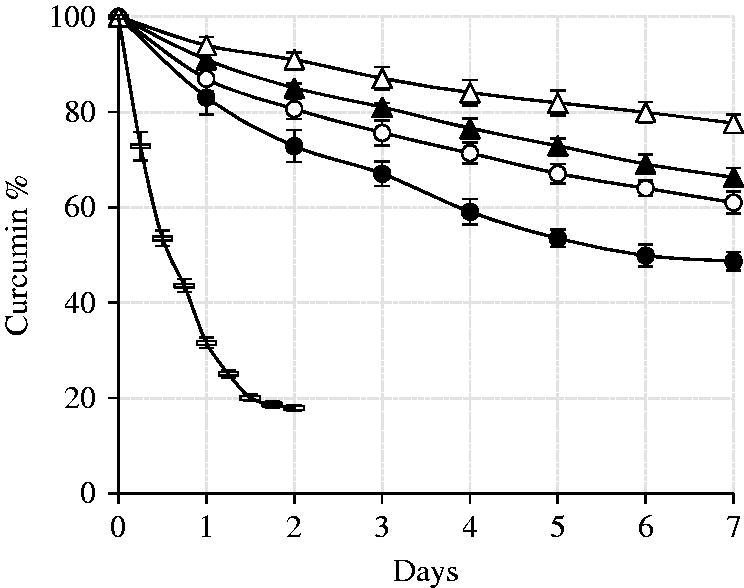
Curcumin degradation in T_3_ (closed circle), T_4_ (open circle), T_5_ (closed triangle), T_5m_ (open triangle) and free aqueous (dash) suspensions. The error bars indicate the standard deviations of three independent experiments.

According to Figure 4, the entrapped curcumin displayed significantly higher stabilities with respect to the free curcumin. The degradation rate constants (*K*) were best fitting to Higuchi kinetic model with normalized intercepts to 100% at day zero (results not shown). The T_5_*_m_* formula proposed the highest stability (*K*_h_= −7.9) comparing to T_3_, T_4_ and T_5_ (*K*_h_ equal to −19.9, −14.5 and −12.0, respectively).

### Cell viability assay

**Figure 5.**
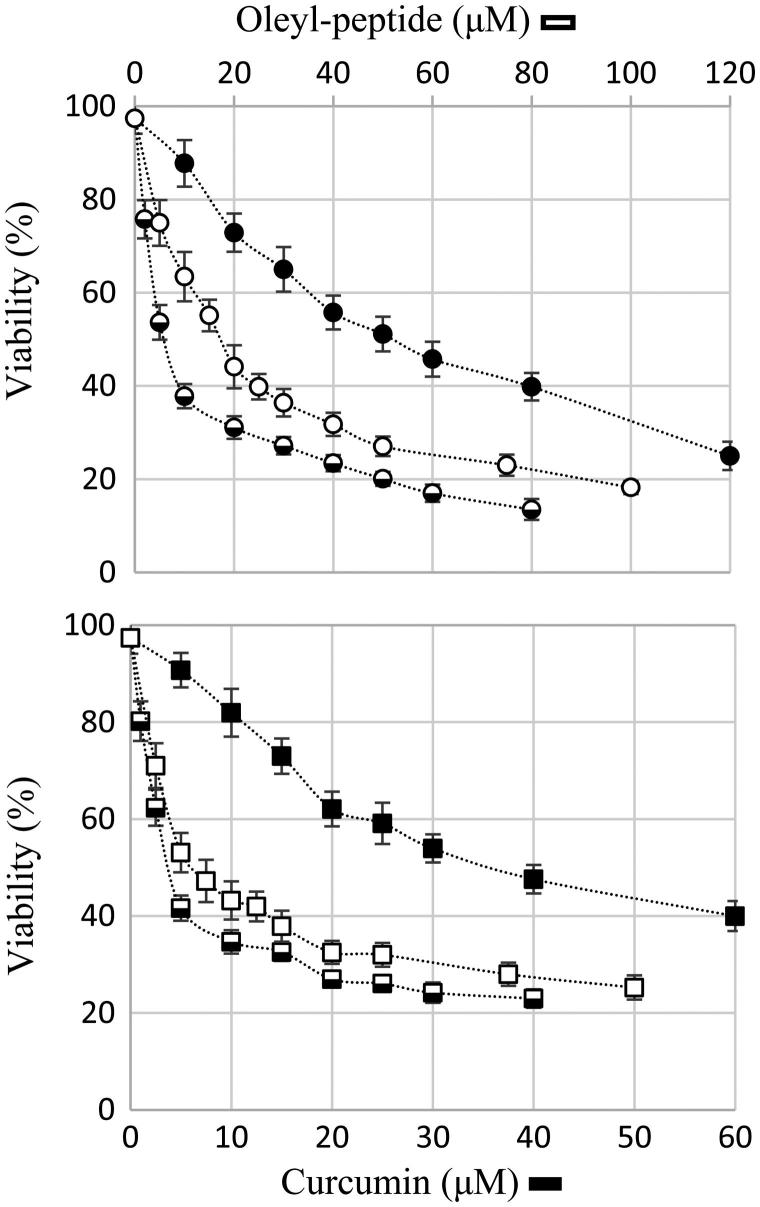
24 h viability assay on MCF-7 (circles) and MDA-MB-468 (rectangles); free aqueous curcumin (closed symbols), T5 liposomes (half-closed symbols) and blank liposomes (open symbols). The error bars display the standard error of the means over three to four independent experiments.

The viabilities in Figure 5 display an average of 3 − 4 independent experiments in MTT assay. The IC_50_ values were calculated from the median-effect plots (Equation 3) of the normalized viability data. The standard errors (SEM) are the standard deviation of the means of three to four replicates. A one-way analysis of variance (ANOVA) with 95% confidence interval (CI) was done in GraphPad Prism (V 6.05). The means of the blank and the curcumin-loaded liposomes were compared with the mean of the free curcumin in Tukey’s multiple comparison test and were reflected in the adjusted *p* values in Table 3.

**Table 3.** IC_50_ values and Tukey’s multiple comparison (one-way ANOVA with 95%CI).

	MCF-7		MDA-MB-468	
Sample	IC_50_ (SEM)^a^ (μM)	Adjusted *p*†	IC_50_ (SEM)^a^ (μM)	Adjusted *p*†
Free CM	26.4 (1.68)	−	37.4 (1.49)	−
Blank LP	18.1 (1.09)	0.0022	15.7 (1.39)	<0.0001
CM – LP	3.8 (0.30)	<0.0001	5.4 (0.44)	<0.0001

a SEM = SD/*n*^½^ with *n* = 4 (independent experiments).

† Adjusted *p* value in Tukey’s multiple comparison test with free curcumin as the control.

CM: curcumin; LP: liposome.

## Discussion

### Curcumin-loaded nanoliposomes

In a study by Ogunsola et al. (2012), the plots in small-angle neutron scattering indicated a direct correlation between the increasing concentrations of Tween and creation of micellar conformations in flexible transfersomes. In the current study, the T_5_ formula contained 27.27% Tween and tended to conform into the liposomes (Figure 3). According to Patel et al. (2009), the ascending ratio of Tween efficiently improved the entrapment of curcumin from T_1_ to T_4_ (Table 1). The slight increase in the loading % in T_5_, however, was presumed to be the direct influence of lecithin on the bilayer chain ordering.

Based on the results of the study by Bernabé-Pineda et al. (2004), the p*K_a_* values of curcumin were determined at 8.38, 9.88 and 10.51 corresponding to the deprotonation of the central β-diketone, and the two phenolic groups in aqueous solutions, respectively. The hydration of the dried lipid-film (Karewicz et al. 2011; Patil & Jadhav 2014) in phosphate buffer at pH 5.7, in the present study, results in increased deionization of curcumin (Cur^0^) and its tendency for passive diffusion across the bilayer. The non-entrapped curcumin in extraliposomal buffer self-associates and precipitates gradually on the container walls. When the external pH is raised to 8, the precipitated curcumin is re-dissolved at a pH close to its p*K_a1_* and transforms mainly to monovalent anionic species (Cur^0^ → Cur^−^ + H_3_O^+^). The curcumin anions, however, are not permeable and reside mostly in the suspending buffer or attached onto the surface of the liposomes. When the pH is descended to 5.7, the major population of the anionic curcumin transforms to neutral species (Cur^−^ + H_3_O^+ ^→ Cur^0^) and penetrates into the lipid bilayer with a lower fraction permeating across the membrane. There is a high possibility that the entrapped curcumin self-associates and precipitates in the internal region or on the interior surfaces when its concentration exceeds the maximum saturated concentration (Tønnesen 2006). Correspondingly, the remaining unentrapped curcumin reassembles in the external buffer and aggregates to large colloidal particles that are separable by membrane filtering. Curcumin binding onto the liposome surface has also been proposed (Huang 2009) and is strongly correlated to the formation of electrostatic and hydrogen bonds (Barry et al. 2009). In a study performed by Zhou et al. (2014), curcumin molecules were suggested to be located near the head groups of sodium bis(2-ethylhexyl) sulfosuccinate (the AOT surfactant), in micellar constructions while in the vesicles, it could deeply penetrate into the more hydrophobic region of the bilayer. The internalization of the reactive keto-enol group within the hydrophobic tails of the amphiphilic sheets resulted in highly reduced flexibility and minimum ionization due to a great reduction of the number of molecules interacting with surrounding water. This phenomenon was also revealed in the experiment by Hung et al. (2008) where the binding of curcumin to lipid bilayers was suggested to affect the functions of membrane proteins by reducing the thickness and the elastic property of the host lipid bilayer. The instantaneous switch in the pH of the suspending buffer, in this experiment, however, significantly promoted the loading % to nearly threefolds in T_5_*_m_*. The current strategy, though is different from the active loading (Gubernator 2011), takes the advantage of the intrinsic slow self-aggregation of neutral species of curcumin within the pH frame of 5 to 8 and results in a pseudo-active loading of low water-soluble drug.

### In vitro analysis: particle size, zeta potential and curcumin stability

Table 2 confirmed a continuous reduction in size with an increase in the mole % of Tween from T_1_ to T_4_. This result is mainly attributed to the surface modifying properties of Tween and is capable of producing highly elastic liposomes (Sharma et al. 2002; Nava et al. 2011). According to Table 2, the surface charge of the oleyl-peptide particles greatly varied with the change in the medium and the pH. The relatively large (absolute) zeta of the peptide particles at pH 7 and 3 and the T_5_ liposomes at pH 7.4 indicated the presence of substantial repulsive forces and better stabilization of the particles at these pH regions. The theoretical isoelectric point of the oleyl-peptide was estimated at around 8.97 (MarvinSketch, 6.1.4). Nevertheless, a deep shift from +20.3 (pH 3) to −25.92 (pH 7.4) supports the specific adsorption of the bivalent phosphate counter ions within the stern layer and a reversal of the sign at the shear plane (Particle sciences, 2012). At considerably low acidic pH, the glutamate counter ions are not subjected to the active adsorption and the full protonation of the histidine and arginine residues accumulates the positive charges at the shear plane.

The degradation kinetics is often influenced by the drug-, vehicle- and medium-related parameters. Among these, extreme importance is given to factors such as the fraction of neutral monomeric curcumin in the intravesicular space, possible changes in internal pH due to external buffer, the membrane-localized curcumin and probable induction of a negative curvature causing the phase transition of the bilayer from gel to rippled gel (pre-transition) or rippled gel to liquid crystalline (transition), facilitated drug release due to the vehicle degradation, and accelerated degradation due to the release of oxidative agents from the lipid carrier. The degradation of the entrapped curcumin, in the current study, was affected by the mole % of Tween: 33.3% reduction in *K_h_* with 9.5% increase of Tween from T_3_ to T_4_. Likewise, when lecithin was added to T_5_ and with the pseudo-active loading in T_5_*_m_*, the *K_h_* decreased 19% and 38%, respectively. In contrast to entrapped curcumin, the free curcumin went through rapid degradation with a biphasic model (Figure 4). Notably, an accelerated degradation of the free curcumin has been reported in phosphate buffer or at 37 °C than in water or at room temperature (Wang et al. 1997; Barry et al. 2009).

### Cellular tests

The haemolytic activity is usually attributed to cationic peptides with cell penetrating and/or antimicrobial properties. The oleyl-peptide was tested up to 10-fold of its experimental concentration. Comparing the haemolysis % in the sample and the negative control, no considerable haemolytic activity was proved (the % of haemolysis at 2 mM oleyl-peptide, equaled 1.3 ± 0.04; data not shown).

The mean doubling time for MCF-7 and MDA-MB-468 was approximately 24–30 h in several recent studies (Sutherland et al. 1983; Watanabe et al. 2001; Chan et al. 2012; Qin et al. 2014) though higher and lower proliferation rates have been reported by other sources due to the cell density (Cos & Sinchez-Barcelb 1995) or the presence of serum components and growth factors in the culture media (Updike et al. 2005; Androutsopoulos et al. 2009), respectively. Moreover, various cellular analyses demonstrated that curcumin and several curcumin analogues induced mitochondrial apoptosis and cell cycle arrest in sub-G_0_-G_1_ (Ramachandran & You 1999; Sun et al. 2012; Kumar et al. 2014), S (Mehta et al. 1997) and G_2_/M (Mehta et al. 1997; Choudhuri et al. 2005; Kang et al. 2014) phases.

The current MTT results for the free curcumin corresponded to several recent analysis reporting an approximate IC_50_ of 25–35 μM in MCF-7 and after 24 h with a colorimetric technique (Prasada et al. 2009; Jiang et al. 2013; Chen et al. 2014; Mohankumar et al. 2014). This is, while other studies represented similar values after 48 h (Zaidi et al. 2011; Kumar et al. 2014) and Ramachandran et al. (2005) and Li et al. (2015) reported an IC_50_ of 78.72 and 70.2 μM after 24 and 72 h treatment of MCF-7 cells with curcumin, respectively. Similarly, the current results for MDA-MB-468, suggested a cytotoxic effect after 24 h incubation, however, different values have been reported in various studies (> 40 μM after 24 h to 9.7 μM after 5 days) due to the dissimilarity of conditions settled in each experiment (Lin et al. 2009; Yadav et al. 2010; Palange et al. 2012; Thulasiraman et al. 2014). The current findings of IC_50_ agreed on the increased susceptibility of both cell lines to the blank liposomes and the encapsulated curcumin. In all the experiments, the cultures of MDA-MB-468 showed greater resistance to drug-induced cell suppression than the cultures of MCF-7 which might be an outcome of the higher metastatic potential of the former cell line.

The blank liposomes also possessed modest cytotoxicity, which may corroborate the hypothesis of potential interactions of the anchored peptide with the membrane receptors such as Nrp-1 or the integrin family. The binding affinity however, is in close correlation with the configuration of the functional groups (Marchini et al. 2012), chirality (Gentilucci et al. 2007), backbone template (Creighton et al. 2006; Banfi et al. 2007), the stereochemistry and the ring size of the cyclic RGD-mimetic peptides (Belvisi et al. 2006), the metabolic stability and the flexibility (Muller et al. 1997; Roxin & Zheng 2012) of the peptide. The moderate cytotoxicity of peptide-conjugated liposomes may also be an effect of the linear structure of the oleyl-peptide subunits, which lack the required disulphide bridge constraints for a thermodynamically stable configuration of the β-turns and bent conformation (Colombo et al. 2002; Patel et al. 2012).

In addition, a narrow difference was seen between the cytotoxicity of the curcumin-loaded and the blank liposomes, which may be due to the probable interferences of the lipid particles with the endocytosis or the exocytosis of MTT-formazan and the cellular activity of the oxidoreductase enzymes (Ahmad et al. 2006; Angius & Floris 2015). According to Angius and Floris (2015), a loss of the viable cells is a probable outcome when the MTT dye accumulates in the intravesicular space or intercalates into the lipid bilayer of the blank liposomes. After endocytosis, the liposomes are merged with acidifying vesicles leading to destabilization of the lipid membrane, release and the degradation of MTT. In contrast, the increased MTT-reduction in the liposome-treated cells may be explained by the increased permeation and accumulation of MTT into liposomes, increased endocytosis and decelerating/blocking the smooth flow of the cell membrane. In the current experiment, an effect of the blank liposomes on MTT endocytosis and degradation in the lysosomes or the MTT entrapment inside the liposomes with a prolonged backward permeation for metabolic reduction is possible. In both cases, a reduction in the cell viability may occur.

Moreover, the localization of curcumin in the bilayer compartment (Hung et al. 2008; Zhou et al. 2014) and its probable displacement or extraction by competing host molecules such as albumin (Kunwar et al. 2006; Basu & Kumar 2014) and other serum proteins needs to be thermodynamically studied. In the presence of competitive forces, the diffused curcumin is more susceptible to premature release from the gel-phase of the liposomes and further adsorption by serum albumin (2.5 × 10^4^ and 6.1 × 10^4^ M^−1^, estimated curcumin binding constants to phosphatidylcholine and human serum albumin, respectively). Nevertheless, the current results suggested an increased cytotoxicity of around sevenfold for curcumin-loaded nanoliposomes compared to aqueous curcumin suspension in both cell lines. The current data also confirmed around 2.9- and 4.8-fold reduced IC_50_ in cultures of MCF-7 and MDA-MB-468, respectively when incubated with encapsulated curcumin than the blank liposomes. The RGD-grafted liposomes and nanoformulations have been studied in different assays and consistently more efficient cellular uptake of the particles (Naik et al. 2012; Wang et al. 2014) with IC_50_ values of around two to sixfold lower than the parent drug-loaded liposomes were reported (Xiong et al. 2005; Loyer et al. 2013; Li et al. 2014).

## Conclusions

A pseudo-active loading mechanism was proposed in film hydration technique. The increased number of permeable species born with a pH interchange between the neutral and alkaline ranges resulted in higher entrapment % comparing to the passive loading. In contrast to the free drug in the aqueous suspensions, the entrapped curcumin displayed a prolonged half-life and reduced degradation. The current study also supported the benefits of incorporating lipopeptides in the liposome construction. The nano-sized liposomes resulted in significant reduction of the IC_50_ values in cellular experiments.

## Future research

Due to the common pitfalls in dialysis technique, such as the membrane absorption of lipophilic compounds, the release kinetics of curcumin from the nanoliposome with the gel permeation technique for separation of the free and the encapsulated curcumin is currently under study. An ambiguous effect of the blank liposomes on MTT endocytosis and its probable interferences in cellular assays need to be studied with precise measurement of the partitioning of MTT and similar tetrazolium salts into the lipid membrane. Other cellular techniques, such as a flow cytometry-based cytotoxicity assay, are currently under study.
